# Unravelling Microarray Patch Performance: The Role of *In Vitro* Release Medium and Biorelevant Testing

**DOI:** 10.1021/acs.molpharmaceut.4c00459

**Published:** 2024-08-28

**Authors:** Maja Railic, Abina M. Crean, Sonja Vucen

**Affiliations:** SSPC, the SFI Research Centre for Pharmaceuticals, School of Pharmacy, University College Cork, College Road, Cork T12 K8AF, Ireland

**Keywords:** dissolvable microneedles, biorelevant *in vitro* testing, loratadine, chlorpheniramine maleate

## Abstract

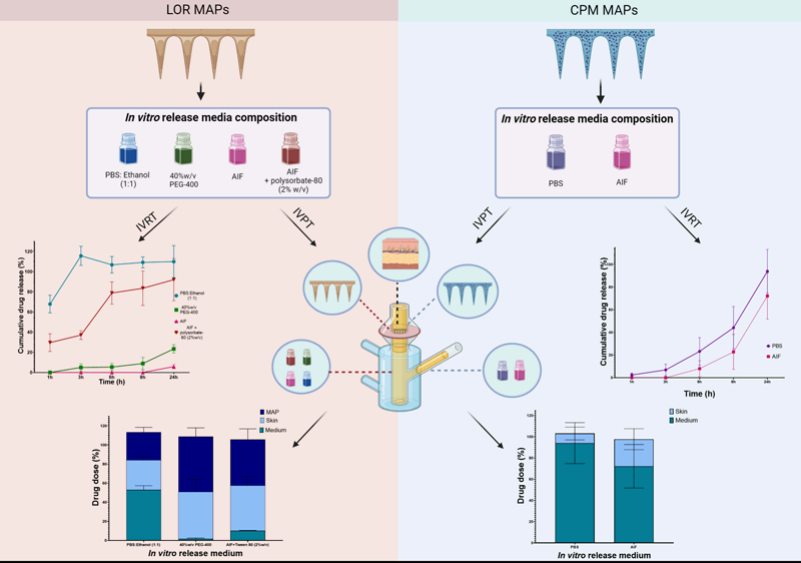

The absence of established protocols for studying the *in vitro* performance of dissolvable microarray patches (MAPs) poses a significant challenge within the field. To overcome this challenge, it is essential to optimize testing methods in a way that closely mimics the skin’s environment, ensuring biorelevance and enhancing the precision of assessing MAP performance. This study focuses on optimizing *in vitro* release testing (IVRT) and *in vitro* permeation testing (IVPT) methods for MAPs containing the antihistamine drugs loratadine (LOR) and chlorpheniramine maleate (CPM). Our primary objective is to investigate the impact of the composition of *in vitro* release media on the drug release rate, penetration through the skin, and permeation into the release medium. Artificial interstitial fluid is introduced as a biorelevant release medium and compared with commonly used media in IVRT and IVPT studies. Prior to these studies, we evaluated drug solubility in different release media and developed a method for LOR and CPM extraction from the skin using a design of experiment approach. Our findings highlight the effect of the *in vitro* release medium composition on both LOR and CPM release rate and their penetration through the skin. Furthermore, we identified the importance of considering the interplay between the physicochemical attributes of the drug molecules, the design of the MAP formulation, and the structural properties of the skin when designing IVRT and IVPT protocols.

## 1 Introduction

Dissolvable microarray patches (MAPs) have emerged as a promising technology for efficient intradermal drug delivery, providing a minimally invasive and patient-friendly approach.^1,2^ These patches incorporate an array of needles designed to effectively penetrate the skin.^3^ They are made from materials that dissolve upon skin penetration, thereby releasing the active pharmaceutical ingredient.^4^ The use of dissolvable MAPs offers numerous advantages, including painless administration, versatile delivery of both small and large drug molecules, minimal skin trauma during insertion, reduced risk of infection, self-administration potential, simplified and safe disposal, and the elimination of the necessity for a “cold chain” to preserve temperature-sensitive medications.^5^

Upon application, MAPs facilitate controlled drug release, allowing the drug to penetrate through different layers of the skin and/or permeate into systemic circulation and reach its designated target site resulting in a localized or systemic therapeutic effect.^6^ Currently, there is a notable absence of compendial assays for testing the performance of MAPs containing drugs and, as a result, guidelines for topical and transdermal drug products are often employed.^7,8^ While the methods provided by these guidelines can be used to evaluate the drug release kinetics from MAP and its penetration through the skin, their direct applicability is impeded by the complex formulations and distinct administration methods of the MAPs. *In vitro* release tests (IVRT) are used to evaluate the rate and extent of release of an active substance from the proposed formulation.^7^ Typically, the test utilizes a non-rate-limiting synthetic membrane and a compatible receptor medium, but in the case of MAPs, the protocol is often customized to accommodate their unique structure. This adaptation entails immersing the MAPs directly in the release medium, maintaining continuous agitation and temperature control, while monitoring drug concentrations at specific time intervals.^9−12^ Conversely, *in vitro* permeation testing (IVPT) is essential to validate the successful penetration of the active substance through the skin and its permeation into the release medium. This is frequently achieved using vertical diffusion cells containing MAPs inserted into a suitable skin model that is in contact with the medium.^13−15^ However, quantification of the drug that penetrated the skin and permeated into the release medium can be a challenging task influenced by various factors such as potentially low drug concentrations, small sample volumes, and the nature of the skin structure itself.^16^ Consequently, further development and optimization of IVRT and IVPT testing protocols tailored specifically to MAPs are necessary for reliable and efficient prediction of MAP performance *in vivo*.

Selecting the appropriate release medium is considered as a fundamental experimental factor affecting drug release rate from semisolid dosage forms and its penetration through the skin.^17,18^ The interplay between physicochemical properties of the drug, chosen formulation, and the composition of *in vitro* release medium becomes even more intricate in IVPT studies when the medium interfaces with the complex skin structure. Nevertheless, the specific impact of these factors on drug release from MAPs and their permeation has not been studied in the structured manner. While phosphate-buffered saline (PBS) at pH 7.4 is the conventional choice for IVRT and IVPT media,^15,19−26^ it may necessitate the addition of cosolvents or surfactants to enhance the solubility of hydrophobic drugs^27^ and ensure compliance with sink conditions.^7^ However, the composition of the medium used for IVPT studies should maintain the integrity of the skin membrane and should not impede drug permeability. Due to the significance of maintaining physiological conditions, the consideration of biorelevant media such as artificial interstitial fluid (AIF) becomes important. An utilization of AIF in IVRT and IVPT studies can closely simulate the physiological environment in which MAPs dissolve. Interstitial fluid has attracted significant interest owing to its inclusion of biomarkers with physiological relevance^28^; however, its utilization for assessing drug release from MAPs has not yet been investigated.

To thoroughly understand the IVPT study findings, it is necessary to utilize reliable methods for measuring the drug within the skin. Various *in vitro* techniques, such as tape stripping, epidermal–dermal heat separation, and Raman spectroscopy can be used to localize drugs in different skin layers and predict their potential for local or systemic therapeutic effects.^29−31^ However, the complex interactions among the drug molecule, skin lipids, and proteins can significantly impede drug recovery efficiency,^32^ making the extraction of the drug from the skin a major challenge. Understanding drug dermatopharmacokinetics (DPK) relies on the inherent physicochemical properties of the drug (e.g., drug solubility, molecular weight, and logP values) that serve as the foundation for developing appropriate testing methods. Prior research has demonstrated variations in the DPK properties of drugs based on their distinct hydrophobicity and molecular weight upon microneedle-mediated delivery, revealing the consequential implication for drug absorption and distribution.^33−35^ Furthermore, the chosen formulation design affects the extent and rate at which the active moiety is available at the site of action.^36^ A study by Moore et al. reported that 83.6% of dissolvable MAPs containing drugs employ polymers as their matrix materials.^37^ The dissolution rate of the polymer matrix can be used to tailor controlled drug release and its DPK profile.^38^ Moreover, an innovative avenue in formulation design arises through the possibility of fabricating MAPs directly from the drug itself.^39,40^ However, factors influencing their DPK have not been investigated yet.

Given the aforementioned factors, this study aims to investigate the impact of the composition of *in vitro* release media used in IVRT and IVPT on the drug release rate and percutaneous absorption through the skin following the application of MAPs. To accomplish this objective, we conducted drug solubility studies and developed an extraction methodology to accurately quantify the drug content within the skin following its release from MAPs. The results have been comprehensively assessed, taking into consideration three main aspects. First, the physicochemical attributes of the two antihistamine drugs, i.e., loratadine (LOR) and chlorpheniramine maleate (CPM), were evaluated based on their differing solubility and lipophilicity. Although topical and transdermal formulations containing LOR and CPM had been investigated,^41−45^ to the best of our knowledge, these antihistamine drugs have not previously been formulated in dissolvable MAPs. Second, the different designs of the MAP formulations, specifically drug-only and polymer-based MAPs, were examined. Lastly, the interplay between the drug characteristics, the formulation design, the skin’s structural properties, and the surrounding release medium was considered.

## 2 Materials and Methods

### 2.1 Materials

CPM and LOR were purchased from KEMPROTEC Limited (UK). Polyvinylpyrrolidone/vinyl acetate PVP/VA (Kollidon VA 64) was donated from BASF SE (Germany). Acetonitrile, methanol, and propanol, all HPLC gradient-grade, were obtained from Sigma-Aldrich (Ireland). Acetic acid glacial and water, both HPLC grade, were purchased from Fisher Scientific (UK). All other chemical reagents were of pharmaceutical grade and used as received.

### 2.2 Fabrication of Drug-Loaded MAPs

Two types of dissolvable MAPs were fabricated using two different antihistamine drugs, LOR and CPM. Molds from polydimethylsiloxane (PDMS) were fabricated and supplied from Tyndall National Institute Cork, Ireland, and used as molding templates for MAPs fabrication. The microneedle array consisted of 25 pyramidal needles (5 by 5 needles) with a height of 500 μm, base width of 333 μm, and array area of approximately 1 cm^2^.

LOR MAPs were fabricated using the micromolding molten drug technique.^39^ Briefly, LOR powder was heated in a vacuum oven (Memmert VO400, Memmert GmbH + Co.KG, Germany) until it reached its melting point of 138 °C and transitioned into a uniform molten mass. PDMS molds were filled with the melted drug under vacuum. After cooling for 8 h, the microneedles were removed from the molds using adhesive tape (3 M Medical Transfer Adhesive, 1524, 3M Ireland Limited, Ireland).

CPM MAPs were fabricated using the micromolding drug solution technique.^46^ Drug formulation, consisting of CPM at a concentration of 70 mg/mL dissolved in a 15% w/v PVP/VA aqueous solution, was dispensed into PDMS molds using a syringe pump (Pump 11 Elite, Harvard Apparatus, UK). Drug solubility studies in the polymer matrix were performed as a part of preformulation studies, and the maximal CPM loading without a risk of recrystallization has been determined using Differential Scanning Calorimetry (DSC) (data not shown). MAPs were dried in PDMS molds for 6 h under vacuum. Subsequently, they were detached from the molds utilizing 3M adhesive tape.

### 2.3 Characterization of Dissolvable MAPs

**Figure 1:**
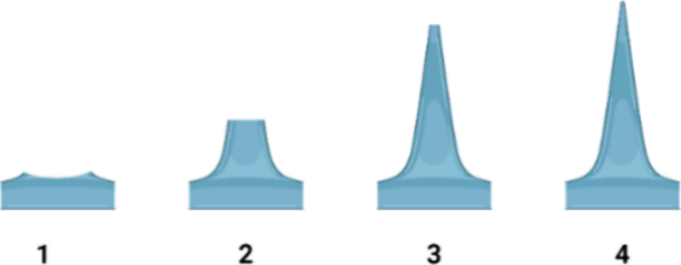
Visual representation of the MAP scoring. Each number represents the scoring of individual microneedle based on their appearance. For example, a perfect MAP with 5 × 5 needles would score 100 based on 25 needles with a score of 4.

The fabricated microneedles were visually characterized for geometry and morphological properties using light microscopy (Olympus SZ61, McCrone Group, Inc., USA). The visual evaluation was conducted based on internal scoring criteria (Figure 1).

The drug content loaded in the microneedles was quantified by dissolving a MAP in 1 mL of appropriate mobile phase and analyzing it using the reverse-phase high-performance liquid chromatography (RP-HPLC) method detailed below (Section 2.5). Ten MAPs of each drug were analyzed and the average content was reported.

Mechanical strength of the MAPs was assessed by performing fracture force measurements.^47^ This entailed the compression of patches utilizing a Texture Analyzer (Stable Micro Systems, UK), with a 35 mm aluminum cylindrical probe (P/35 probe). The velocities were adjusted as follows: 1.5 mm/s for the pretest phase, 0.1 mm/s for the test phase, and 10 mm/s for the post-test phase. The trigger force was 0.06 N. The resulting pressure applied to the needles during displacement was recorded. Three replicates of both LOR and CPM MAPs were analyzed and the average fracture force was reported.

Skin staining studies were performed to visualize the insertion capability of the microneedles. For the experimental setup, fresh porcine ears were procured and stored at −80 °C. Prior to the experiment, the ears were thawed slowly in a refrigerator for 24 h. The skin samples were prepared by removing hair and eliminating subcutaneous fat. This process was performed using a razor and forceps, ensuring the preservation of the skin barrier integrity. LOR and CPM MAPs were inserted into the skin using an in-house designed applicator with a compressive force of 25 N. Subsequently, 20 μL of crystal violet dye was administered over the area where the patch had been applied. After 30 min, tape stripping was performed until the grid of indentations on the skin became visible, which then underwent imaging under a microscope.

### 2.4 Drug Release from MAPs and Skin Permeation

#### 2.4.1 Pretest Screening

Pretest screening involved drug solubility studies to confirm sink conditions for IVRT and IVPT, as well as the development of methods for drug extraction from the skin and transepidermal water loss (TEWL) measurements, both prerequisites for IVPT studies.

##### 2.4.1.1 Drug Solubility Studies

The solubility of the drugs in various release media proposed for their respective IVRT and IVPT studies was measured by using a shake-flask method. The composition of *in vitro* release media for LOR and CPM was determined based on previous IVRT and IVPT studies conducted with other transdermal and topical dosage forms.^41−45,48^ Furthermore, the solubility of LOR and CPM in AIF was also determined, utilizing the composition described by Bretag (Table S1, Supporting data).^49^ The potential for enhancing LOR solubility was investigated by the addition of polysorbate-80 to AIF. Therefore, LOR solubility was assessed in four different media: PBS:ethanol (1:1), 40%w/v PEG-400, AIF at pH 7.4, and the same AIF with the addition of polysorbate-80 (2%w/v). Similarly, the solubility of chlorpheniramine maleate was evaluated in two media: PBS and AIF, both at pH 7.4.

Excess drug was added to each medium and continuously agitated using an orbital shaker (Stuart SSM1 Orbital Shaker, Keison Products, UK) at 37 °C. Samples were withdrawn at predetermined intervals (24, 48, and 72 h) and analyzed using RP-HPLC to determine the drug equilibrium solubility. Three replicates were generated for each media.

##### 2.4.1.2 Extraction of the Drug from the Skin

A design of experiments (DoE) approach was used to assess the impact of individual factors and their interactions on the drug recovery from the skin. Porcine skin was defrosted and prepared as described Section 2.3. Minitab software (State College, USA) was utilized to generate a set of experiment runs. Experimental factors and levels for LOR and CPM individual DoEs are shown in Table 1 with further details provided in the Supporting Information (Tables S2 and S3, Supporting data). The identification of key factors influencing the drug extraction method was informed by previous investigations and the physicochemical properties of the proposed drug molecules.

**Table 1:** Factors and Levels Incorporated in a Full-Factorial Design to Study Extraction of Each Drug from *Ex Vivo* Porcine Skin

**drug**	**factors**	level 1	level 2	level 3
**LOR**	extraction solvent	methanol	methanol:acetonitrile (ACN)(70:30)	n/a
	temperature	55 °C	uncontrolled room temperature	n/a
	number of homogenization cycles	1	2	n/a
**CPM**	extraction solvent	methanol:water(80:20)	ethanol:water(70:30)	Propanol:Water(70:30)
	additional grinding step	yes	no	n/a

The method development involved intradermally injecting a known concentration of drug as a bolus into a 1 cm^2^ area of porcine skin, which represented a surface area of individual MAP. After ensuring uniform distribution, the skin sample was cut into small pieces. For CPM samples, an additional grinding step was conducted by placing pieces of the skin in a mortar along with the extraction solvent and then grinding the mixture for 2 min using a pestle. The skin samples were then homogenized in the gentleMACS C-tubes with the proposed solvent using gentleMACS Dissociator (Miltenyi Biotec, USA). Subsequently, the LOR samples underwent temperature investigation by heating the C-tubes in a water bath for 30 min. Both CPM and LOR samples were subjected to centrifugation using the MICRO 200R centrifuge (Andreas Hettich GmbH & Co. KG, Germany) followed by the drug quantification using RP-HPLC.

The optimal combinations of experimental factors to maximize extraction of each drug from the skin was obtained by analysis of the results. These conditions were validated by conducting skin extraction experiments for each drug in quadruplicate.

##### 2.4.1.3 TEWL Measurements

TEWL measurements were performed to investigate the influence of the proposed *in vitro* release media on skin integrity. Following the skin preparation described in Section 2.3 above, skin samples were mounted on vertical Franz diffusion cells (PermeGear, USA) having the receptor compartments filled with the respective media. To achieve a skin surface temperature of 32 °C, a water bath system was used. TEWL was measured using a VapoMeter SWL4001TJ (Delfin Technologies Ltd., Finland) after 30 min and 24 h of incubation. Ambient relative humidity and temperature were recorded and maintained constant for each measurement. The skin surface was ensured to be free of residual moisture before recording TEWL. Each measurement was repeated three times for each skin sample. The data were expressed as the mean and standard deviation.

#### 2.4.2 IVRT Studies

IVRT studies involved submerging LOR and CPM MAPs into the specified release media, the composition of which is outlined in Section 2.4.1.1. The volume of the release media was tailored to meet the sink conditions criteria, ensuring that it exceeded 3–10 times the saturation volume for each respective medium. Samples were incubated at 37 °C in a water bath with continuous agitation at 100 rpm. Investigations into drug release profiles were conducted over a 24 h period for LOR, except for a 3 h duration in PBS:ethanol (1:1) media and a 1 h duration for CPM. Differences in the duration for the release studies were due to release rates in different media and to ensure complete release. At predetermined intervals, samples were withdrawn and replaced with fresh release medium. These withdrawn samples were subjected to a drug content analysis using RP-HPLC. MAP drug release in each medium was tested in triplicate.

#### 2.4.3 IVPT Studies

IVPT studies were conducted using vertical Franz diffusion cells (PermeGear, USA). The porcine skin was handled and prepared in accordance with the aforementioned procedures (Section 2.3). Skin samples were then cut into the appropriate pieces for use in the vertical diffusion cell setup.

The skin diffusion area was 1 cm^2^. To achieve a skin surface temperature of 32 °C, a water bath system was used to heat the receptor compartment to 37 °C. The receptor chamber was filled with designated release medium detailed in Section 2.4.1.1. MAPs were inserted into the prepared porcine skin samples using an in-house applicator (25N) and secured in the donor compartment using adhesive tape. Sampling ports were covered with Parafilm M (BRAND GMBH, Germany) to prevent medium evaporation. At defined intervals, samples were extracted from the receptor compartment and replaced with an equal volume of the release medium. RP-HPLC was utilized to determine the drug concentration in the receptor compartment. After the last time point (24 h), MAPs were removed and dissolved in a mobile phase to determine the remaining drug content. The drug concentration in the skin was quantified using the optimized previously developed method (Section 2.4.1.2).

### 2.5 HPLC Analysis

Drug quantification was performed using RP-HPLC (Agilent 1200, Aglient Technologies, USA) with a C18 column (4.6 mm × 150 mm, 5 μm packing, ZORBAX, Agilent Technologies, USA). For LOR analysis, the HPLC method reported by Spac et al.^50^ was used with a mobile phase of 50 mM acetate buffer solution and methanol (15:85) at a flow rate of 1 mL/min and UV detection at 248 nm (calibration range: 31.25–1000 μg/mL, R^2^ = 0.9954, LOD = 0.03 ng/mL, LOQ = 0.1 ng/mL). CPM was detected at 265 nm using a mobile phase of 0.05 M ammonium acetate and acetonitrile (60:40) with pH 3.5, at a flow rate of 1 mL/min^51^ (calibration range: 31.25–1000 μg/mL, *R*^2^ = 0.9993, LOD = 0.05 ng/mL, LOQ = 0.14 ng/mL).

### 2.6 Statistical Analysis

The data underwent analysis using GraphPad Prism 8.4.3 (GraphPad Software Inc., USA) and MiniTab 20 software (State College, USA). This involved one-way and two-way analysis of variance (ANOVA) as well as an unpaired Student’s *t* test for pairwise comparisons. Post hoc comparisons were performed using Tukey’s HSD and Sidak tests. A significance level of *p* < 0.05 was applied in all cases.

## 3 Results

### 3.1 Characterization of LOR and CPM MAPs

**Figure 2:**
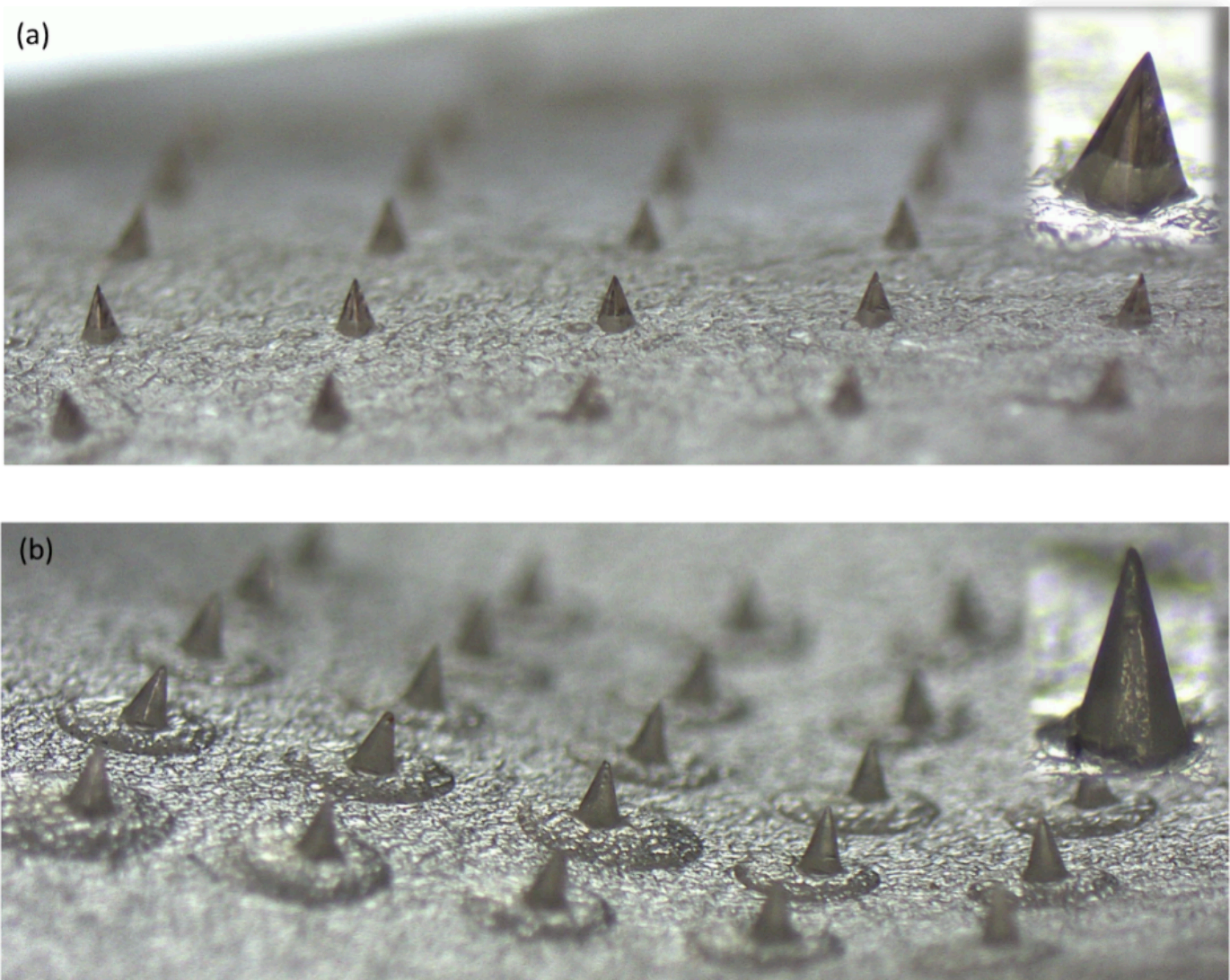
Representative micrographs of (a) LOR MAP 5 × 5 array and (b) CPM MAP 5 × 5 array, with inserted image of single microneedles.

**Figure 3:**
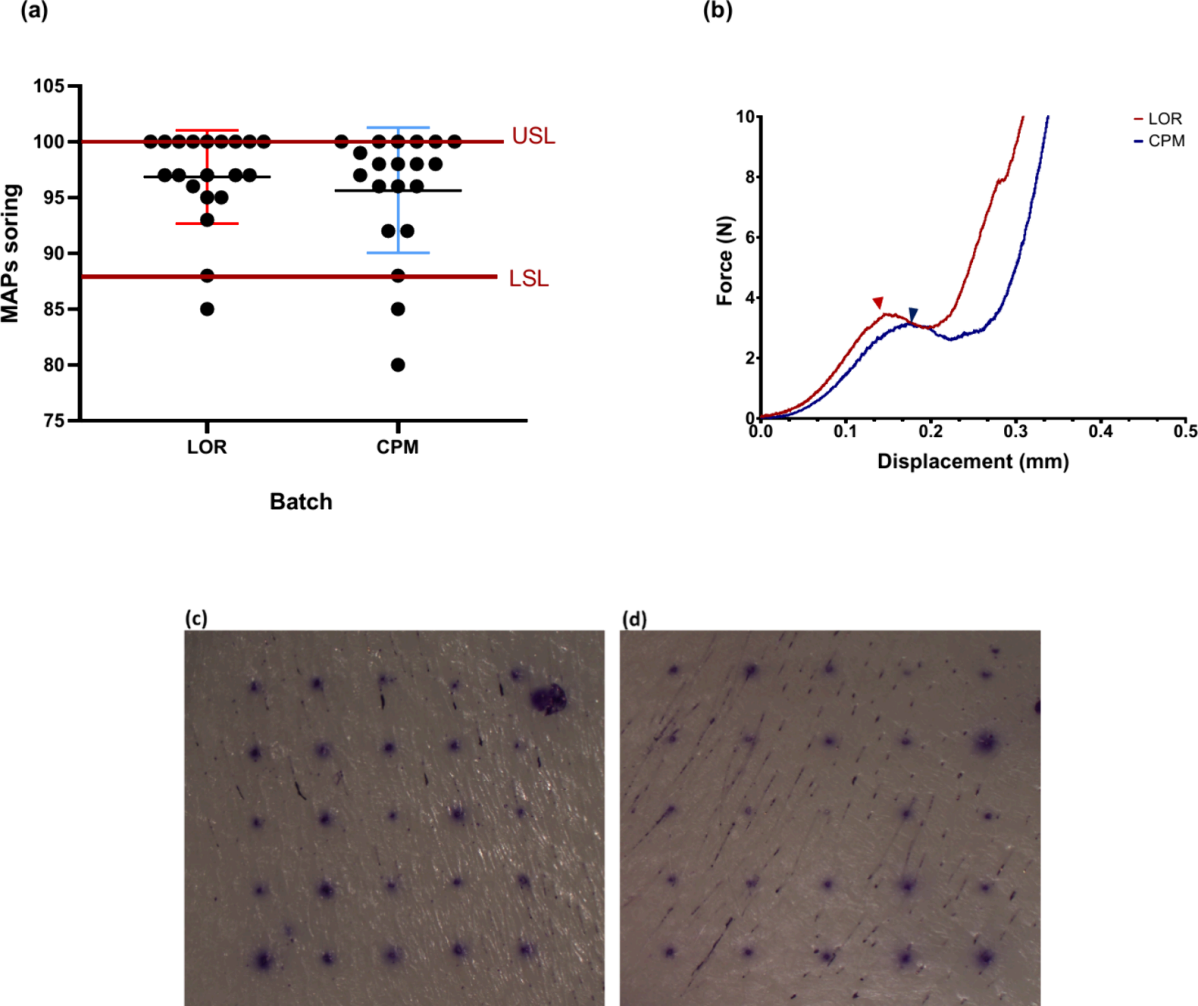
(a) The average MAPs scores achieved for LOR and CPM MAPs were 96.8 ± 4.07 and 95.7 ± 5.44 (*n* = 20), respectively. Maximum achievable score was 100 and represented the upper specification limit – USL. The score of 88 represented a lower specification limit – LSL, and was based on pharmacopeia requirements for drug content uniformity of single dose transdermal patches; (b) fracture forces of LOR (red) and CPM (blue) MAPs were 3.8 ± 0.08 and 3.2 ± 0.14 N (*n* = 3), respectively. Arrows indicate breakage points. Porcine skin after treatment with (c) LOR MAP and (d) CPM MAP and staining with crystal violet.

LOR and CPM MAPs were successfully fabricated by using micromolding molten and drug solution methods, respectively (Figure 2). Individual patches were visually assessed, and the mean score obtained for LOR and CPM MAPs was 96.9 ± 4.07 and 95.7 ± 5.44, respectively (Figure 3a). Drug load capacity in LOR MAPs, as expected, was higher than in CPM MAPs (0.5 ± 0.09 and 0.1 ± 0.02 mg per patch, respectively) considering that LOR MAPs are composed of drug alone. Mechanical test results revealed the fracture forces of the LOR and CPM MAPs to be 3.8 ± 0.08 and 3.2 ± 0.14 N, respectively (Figure 3b). The fabricated MAPs exhibit sufficient mechanical strength that was above the reported threshold necessary for skin insertion without fracturing (0.098 N/needle or 2.45N/MAP containing 25 microneedles), as found in previous studies.^52,53^ Following MAP application, the formation of microchannels in the skin can be detected by observing their capacity to absorb crystal violet.^54^Figure 3c,d displays porcine skin images stained with crystal violet following treatment with LOR and CPM MAPs, respectively, showing the formation of microchannels.

### 3.2 Drug Release from MAPs and Skin Permeation

#### 3.2.1 Pretest Screening

**Figure 4:**
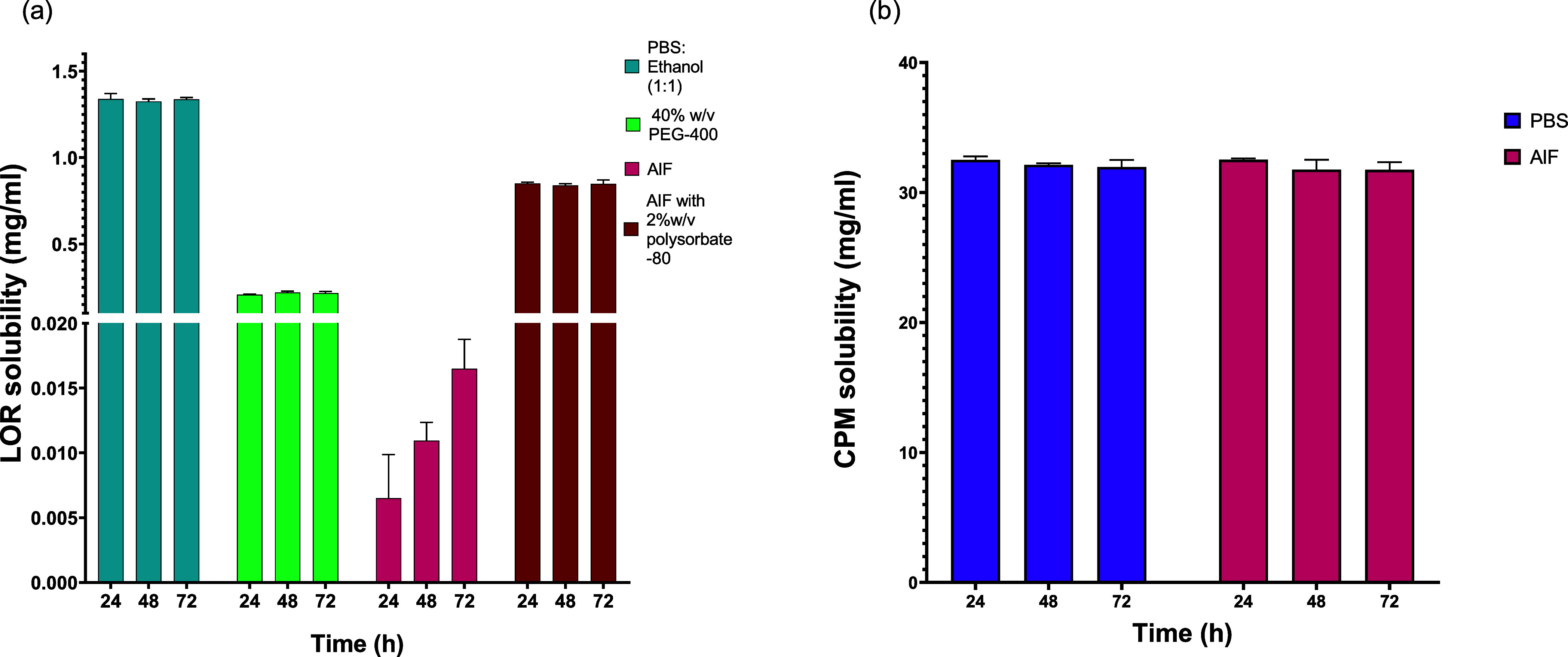
(a) LOR solubility studies in PBS:ethanol (1:1), 40% w/v PEG-400, AIF, and AIF with the addition of polysorbate-80 (2%w/v), (*n* = 3); (b) CPM solubility studies in PBS and AIF (*n* = 3). No statistically significant differences were observed among various time points for both drugs (*p* = 0.6540 and *p* = 0.7893 for LOR and CPM, respectively, two-way ANOVA). Y error bars indicate the standard deviation.

Solubility studies evaluated the drugs’ solubility in the designated release media and verified their compliance with sink conditions criteria for IVRT and IVPT studies, Figure 4. LOR solubility was highest (1.33 ± 0.01 mg/mL) in medium containing PBS and ethanol (1:1). In contrast, the solubility of LOR in 40%w/v PEG-400 was notably lower (0.22 ± 0.01 mg/mL). The inclusion of 2%w/v polysorbate-80 in AIF significantly enhanced the solubility of LOR, resulting in a concentration of 0.87 ± 0.01 mg/mL. In the absence of polysorbate-80, the drug solubility markedly decreased to 0.011 ± 0.002 mg/mL (Figure 4a). Considering the volume of the Franz diffusion cell (7 mL) and the dose of LOR loaded per patch (0.5 ± 0.09 mg), it is evident that the utilization of AIF without polysorbate-80 would fail to maintain the requisite sink conditions for IVPT studies.

Conversely, CPM demonstrated comparable solubility levels in both proposed media. The solubility of CPM was found to be 31.9 ± 0.45 and 31.7 ± 0.48 mg/mL in PBS and AIF, respectively (Figure 4b). These findings indicate that both PBS and AIF can be suitable media for conducting IVPT studies in our experimental setup, as sink conditions can be maintained throughout the experiments.

**Figure 5:**
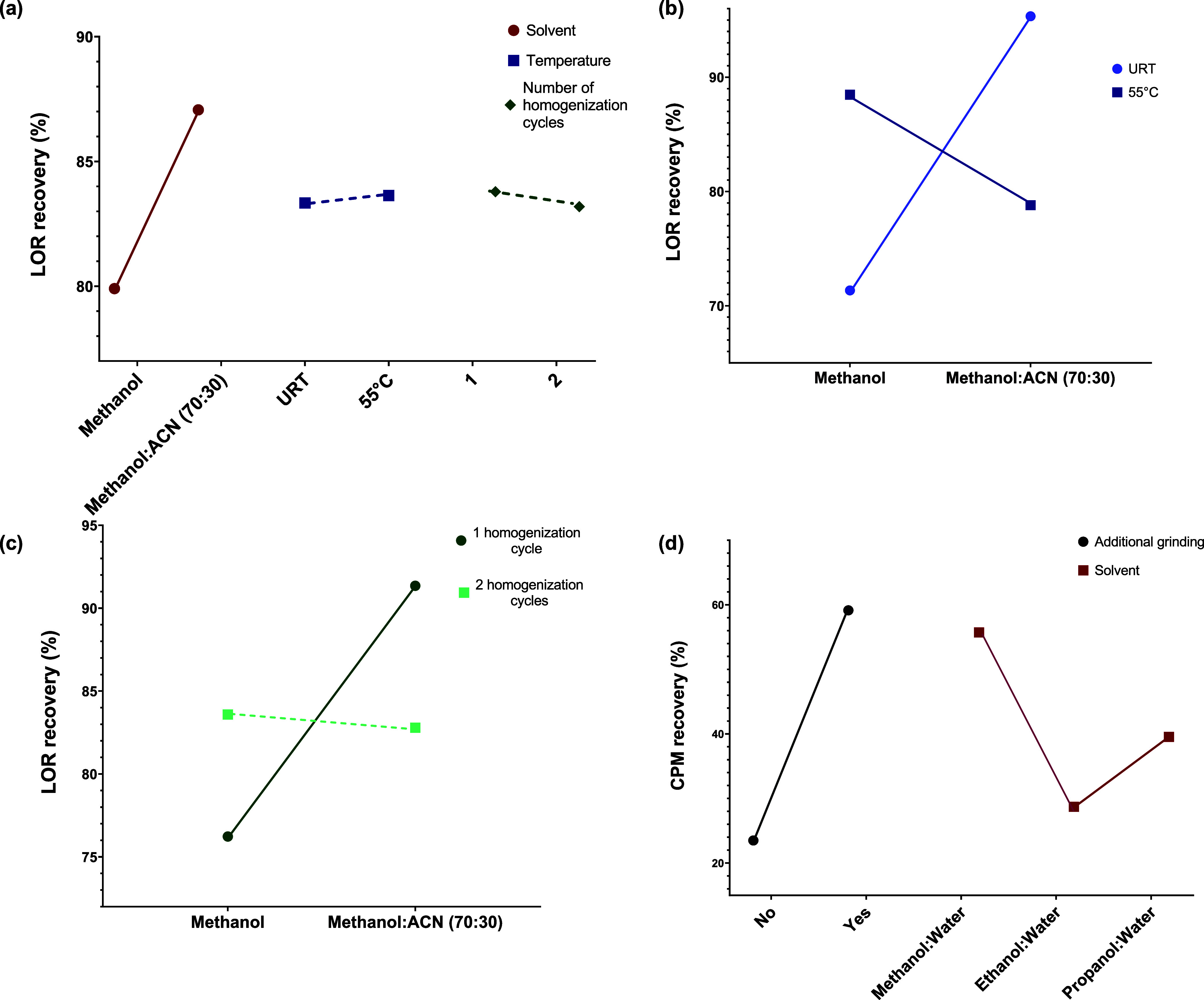
(a) The extraction solvent had the most significant impact on LOR extraction efficiency (*p* = 0.049), while temperature (*p* = 0.925) and homogenization cycle (*p* = 0.851) showed nonsignificant effects (two-way ANOVA, MiniTab). Interaction plots presenting (b) solvent-temperature and (c) solvent-homogenization cycle indicate that, with methanol, both elevated temperature and second homogenization cycle lead to a higher percentage of LOR recovery from the skin, whereas no such increase is observed with a mixture of methanol and ACN; (d) both the additional grinding step (*p* < 0.001) and extraction solvent (*p* = 0.009) significantly affected CPM recovery from the skin (two-way ANOVA, MiniTab). Solid lines represent significant factors and interactions, while dashed lines present nonsignificant factors and interactions. URT denotes uncontrolled room temperature.

Through the application of DoE analysis, we identified the key factors that exert significant influence on the skin extraction of the proposed drug compounds. We discovered that the choice of extraction solvent significantly emerges as the most influential factor impacting the average percentage of LOR recovery from the skin (Figure 5a). Furthermore, our analysis revealed statistically significant interactions between solvent-temperature and solvent-homogenization cycle (Figure 5b,c) (Figures S1 and S2, Supporting Information). Using a binary mixture of methanol and ACN as the extraction solvent at room temperature, along with a single homogenization cycle, resulted in the highest LOR recovery efficiency. The method validation was performed using four replicates, resulting in LOR recovery of 93.9 ± 6.02%.

In the case of CPM, DoE analysis emphasized the significant impact of an added grinding step in skin extraction, as presented in Figure 5d. Moreover, the significance of the solvent as a secondary factor was also evident for CPM. The interaction between these two factors exhibited no significant impact on drug recovery rates (Figure S2, Supporting Information). Among the solvents investigated, the highest CPM recovery was attained by employing a mixture of methanol and water (80:20) along the additional grinding step by using a mortar and pestle after skin cutting. The achieved drug recovery was 83.1 ± 0.43% of the injected CPM amount. To validate the method, four replicates were conducted using these conditions, resulting in an average CPM recovery of 77.6 ± 5.67%.

Following a 30 min incubation, TEWL values for PBS:ethanol (1:1), 40%w/v PEG-400, AIF with polysorbate-80 (2% w/v), PBS, and AIF were 5.13 ± 0.63, 4.67 ± 1.18, 3.97 ± 1.13, 2.97 ± 0.34, and 2.33 ± 0.41 g/m^2^ h, respectively. After 24 h, only incubation with PBS:ethanol (1:1) exhibited a significant TEWL value increase of 11.3 ± 1.79 g/m^2^ h (*p* = 0.0049, unpaired *t* test), slightly surpassing the standard limit of 10 g/m^2^ h.^55^ According to recent findings by Schoenfelder et al., TEWL values should ideally be below 20 g/m^2^ h, indicating that the skin maintained acceptable barrier properties after 24 h despite the TEWL increase.^56^ The addition of nonionic surfactants (polysorbate-80 and PEG-400) in proposed concentrations did not impede skin integrity, which aligns with previous findings.^57^

#### 3.2.2 IVRT Studies

**Figure 6:**
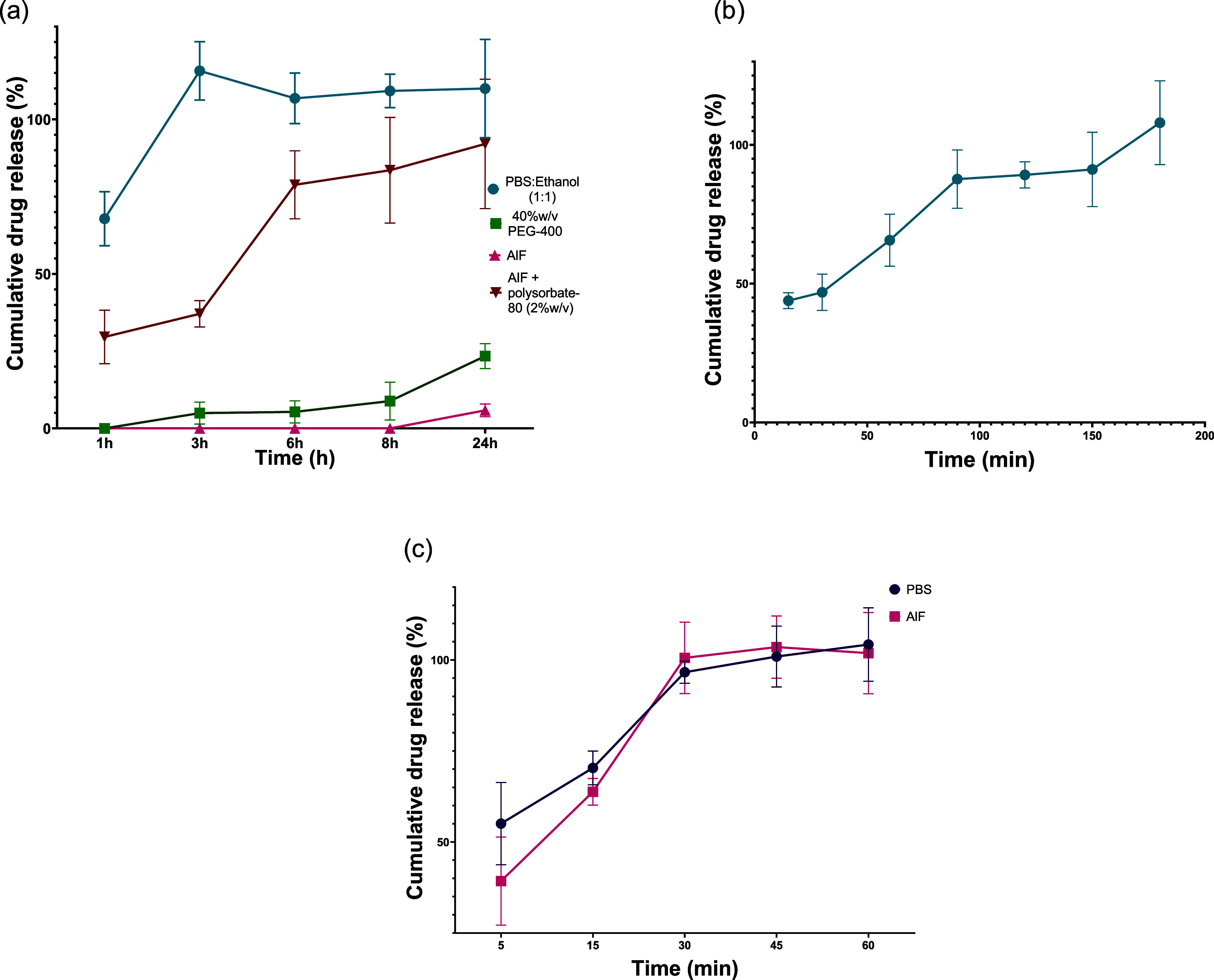
LOR *in vitro* release profile (a) in different release media over 24h (*n* = 3) and (b) in PBS:ethanol in the first 3 h (*n* = 3). Drug release was significantly different among different release media (*p* < 0.0001, two-way ANOVA); CPM (c) release showed no significant difference between PBS and AIF (*n* = 3) (*p* = 0.2750, two-way ANOVA). Y error bars indicate standard deviation.

IVRT studies were performed to examine the release profile of LOR and CPM in different release media, Figure 6.

The results revealed significant differences in LOR release between different release media (*p* < 0.0001, two-way ANOVA) (Figure 6a). The most rapid release occurred when LOR MAPs were immersed in media containing a 1:1 mixture of PBS and ethanol, with an initial burst release of 67.85 ± 8.74% LOR within the first hour, reaching 100% release by 3 h. Further investigation with more frequent sampling points over a 3 h period demonstrated over 40% LOR release within the first 30 min and more than 90% by 150 min (Figure 6b). LOR exhibited almost complete release in AIF with the addition of 2%w/v polysorbate-80 (92.06 ± 20.91% of the drug dose after 24 h), although its release rate in this medium was significantly slower from 1 to 8 h compared to PBS:ethanol (two-way ANOVA, *p* ≤ 0.05). In contrast, LOR release in 40%w/v PEG-400 and AIF media was notably slow, particularly in AIF, where only 5.85 ± 2.98% of LOR was released after 24 h.

CPM release profile from CPM MAP showed no significant differences between the two release media studied at each time point, as confirmed by a two-way ANOVA (*p* = 0.2750). An initial burst release was observed, with 55.06 ± 11.29% and 39.29 ± 12.09% of the drug dose being released within just 5 min in PBS and AIF, respectively. The drug completely dissolved withing 30 min, as illustrated in Figure 6c.

#### 3.2.3 IVPT studies

The influence of the release medium on drug penetration through the skin after MAP application was assessed by quantifying the drug permeated in the proposed medium and its skin deposition in IVPT studies over a 24 h period. Furthermore, the remaining drug content on MAP after each experiment was determined.

**Figure 7:**
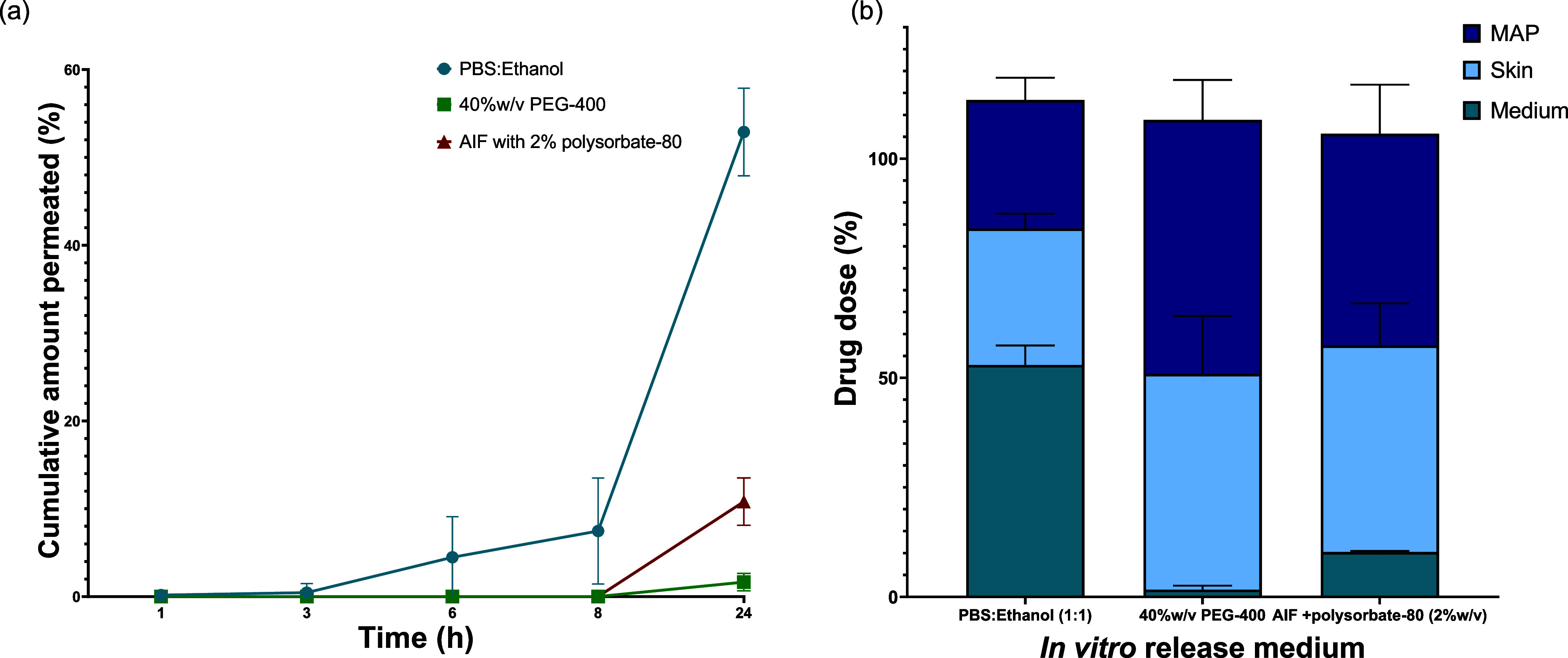
(a) *In vitro* permeation profile of LOR in different release media (*n* = 5). A statistically significant effect of the release media was observed at the 24 h time-point (*p* < 0.001, two-way ANOVA); (b) distribution of LOR between MAP, skin, and release media varied with different release media used (*n* = 5). Y error bars indicate standard deviation.

The cumulative amount of LOR permeated in PBS:ethanol (1:1), 40%w/v PEG-400, and AIF with the addition of polysobate-80 (2 wt %) is presented in Figure 7a. The AIF medium was not included in this study since it did not meet the criteria for sink conditions using our experimental setup. Our findings demonstrate significant variations in the permeation patterns of LOR between the different media used (*p* < 0.001). Further analysis using Tukey’s multiple comparison test revealed significant differences in the percentage of cumulative LOR amount permeated after 24 h across all three media. Specifically, the percentage of cumulative amount permeated was 52.89% ± 4.47, 1.65% ± 0.89 and 10.26% ± 0.24 in PBS:ethanol (1:1), 40%w/v PEG-400 and AIF with 2%w/v polysorbate-80, respectively (*p* < 0.0001 between PBS:ethanol (1:1) and 40%w/v PEG-400, *p* < 0.0001 between PBS:ethanol (1:1) and AIF with polysorbate-80 (2%w/v), and *p* = 0.0019 between 40%w/v PEG-400 and AIF with polysorbate-80 (2%w/v)). After the 24 h experiment, a two-way ANOVA (*p* = 0.0553) indicated no significant differences in drug content in the skin across the three release media conditions. However, significant variations were observed in the residual LOR content on the MAP under different release media conditions (*p* < 0.0020) (Figure 7b). Supporting data include box plots illustrating the mean drug amounts in the skin and tape across different media conditions (Figure S3, Supporting Information).

**Figure 8:**
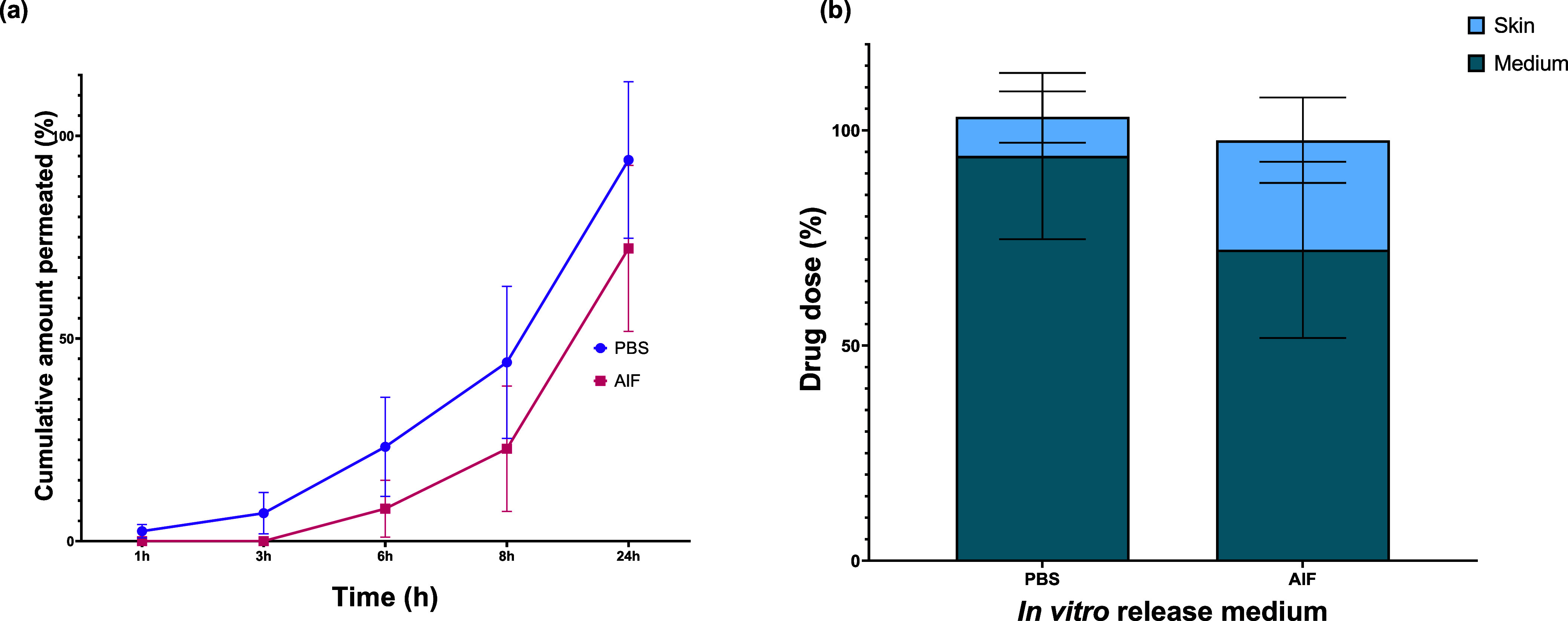
(a) CPM *in vitro* permeation profile in different release media (*n* = 5). Statistically significant effect of the release media was observed at last time point (24h) (*p* = 0.0474, two-way ANOVA); (b) the predominant percentage of the drug dose is observed in the release medium, with elevated levels of CPM detected in the skin when AIF is employed as the *in vitro* release medium. Y error bars indicate standard deviation.

The cumulative permeation profiles of CPM in PBS and AIF are presented in Figure 8a. Specifically, after 24 h, the percentage of cumulative amount permeated was 94.04 ± 19.31% in PBS and 72.23 ± 20.47% in AIF. Subsequent Sidak posthoc tests indicated that the cumulative amount of CPM permeated was significantly different after 24 h in PBS and AIF (*p* = 0.0474). The skin used in these studies was analyzed for CPM content, and an unpaired *t-*test analysis demonstrated a significant difference in the amount of CPM present when using PBS and AIF as release media (*p* = 0.0220) (Figure S4, Supporting Information). Interestingly, no traces of the drug were detected on the MAP after using either release medium (Figure 8b).

## 4 Discussion

The *in vitro* assessment of DPK holds a pivotal role aimed at ensuring the identity, strength, quality, purity, comparability, and overall performance of dermal drug products.^58^ Among these tests, IVRT and IVPT have become fundamental tools in pharmaceutical drug development.^59^ Successful design of these tests includes consideration of numerous experimental factors such as the composition and pH of the release medium, stirring speed, temperature, skin model, duration of the experiment, and more. The choice of *in vitro* release medium was identified as the most critical parameter influencing drug release from topical dosage forms.^17,60^ To the best of our knowledge, the impact of the *in vitro* release medium on the drug release from MAP and its subsequent skin penetration has not been previously explored. Hence, we investigated how the composition of *in vitro* release media affects the release rate and skin penetration of the LOR and CPM in MAP formulations.

According to the Biopharmaceutics Classification System (BCS), LOR is classified in BCS Group II, demonstrating low solubility (categorized as practically insoluble) but high permeation properties while CPM falls into BCS Group I, exhibiting high solubility (categorized as freely soluble) and high permeability. Differences in LOR and CPM solubilities highlight the different considerations in the selection of appropriate drug release media for each drug molecule. To simulate physiological conditions, this study introduces AIF as an *in vitro* release medium. Low LOR solubility in AIF was enhanced by the addition of 2 wt %/w polysorbate-80 and established sink conditions within the utilized Franz cell experimental setup. LOR solubility, both in the AIF containing 2%w/v polysorbate-80 and in PBS:ethanol (1:1), coincided with an accelerated and more extensive release rate in IVRT. Conversely, selecting an appropriate *in vitro* release medium for testing hydrophilic molecules such as CPM posed fewer challenges owing to their higher solubility in PBS. PBS is a commonly used medium due to its pH and osmotic balance, which closely resemble physiological conditions. In contrast, AIF contains various additional salts and sugars that could influence the drug release. Nonetheless, our findings showed similar CPM solubilities in PBS and AIF as well as similar drug release profiles in IVRT studies. In addition to drug solubility, the formulation strategies employed in manufacturing MAPs can significantly influence drug release rates. While CPM MAPs utilized PVP/VA as a polymer matrix material, already known for its fast dissolution rate,^61−63^ LOR MAPs consisted solely of the drug. Given LOR’s highly lipophilic nature and the absence of a polymeric material, the LOR MAPs exhibited a relatively slower drug release pattern compared to CMP MAPs. Furthermore, *in vitro* release media composition, coupled with drug physicochemical properties and formulation choice, can greatly influence the understanding of underlying drug release mechanisms.^64,65^ Typically, these mechanisms are discerned using mathematical models like Higuchi,^66^ Korsmeyer–Peppas,^67^ Hixon–Crowell,^68^ and Weibull.^69^ Yet, these models may not fully capture the complexities of dissolvable MAPs, as they overlook microneedle geometry and formulation design. This highlights the need for further research to develop models that accurately reflect the release kinetics specific to dissolvable MAPs. It is noteworthy that sink conditions are usually employed to simulate scenarios where the released drug is rapidly absorbed or eliminated, thereby emphasizing dissolution as the rate-limiting step. However, the use of dissolvable MAPs and the consideration of very small volumes of interstitial fluid in the skin pose challenges to adhering to traditional sink conditions, raising questions about their relevance.

The meaningful interpretation of IVRT results is closely linked to the subsequent IVPT analysis, as these combined investigations provide a holistic perspective on the performance of MAPs. In our IVPT studies, lower concentrations of both drugs were detected in the release medium compared with the IVRT studies, suggesting interactions between the drug and surrounding skin environment. The primary physicochemical parameter that characterizes the affinity of small drug molecules for the skin is their lipophilicity, typically expressed by the logP value. The impact of lipophilicity on the bioavailability of drugs after percutaneous administration via MAPs has been previously explored in the literature and revealed a decrease in systemic bioavailability with increasing logP value of the drug.^33,35^ Considering the logP values of our drugs (5.2 and 3.62 for LOR and CPM, respectively),^70^ both drugs were expected to predominantly remain in the skin and potentially achieve a local therapeutic effect. However, CPM’s amphiphilic nature, with a hydrophobic ring and a charged cationic amino group, makes it hydrophilic,^44^ impacting its permeation due to increased solubility in the release medium. Furthermore, the interaction between the drug and different skin layers can differ due to the unique characteristics of each layer. Unlike the epidermis, the dermis does not manifest a formidable barrier for hydrophilic molecules’ passage.^71^ Given the design of our MAPs, which are expected to reach the upper dermis layer,^72^ the increased amount of CPM in the medium can be attributed to this difference in drug permeability. Moreover, understanding the interaction between the drug molecule and skin layers is crucial when developing a reliable extraction methodology, as evidenced by our findings. In addition to a suitable extraction solvent capable of disrupting the skin barrier and dissolving the proposed drug molecule, an additional grinding step proved beneficial in achieving higher recovery rates for hydrophilic molecules, primarily retained in aqueous compartments such as intercellular spaces.^73^

The distribution of drug content between the medium, skin, and MAP following the IVPT studies demonstrated significant variations with regard to the choice of *in vitro* release media. As previously mentioned, the addition of cosolvents and surfactants becomes crucial when addressing poorly soluble drugs. Potential interactions between the release medium and the underlying layers of the suggested skin model can result in the dissolution of specific skin compounds, ultimately altering the drug’s penetration properties through the skin,^74^ and therefore require thorough consideration in IVPT testing. For example, ethanol is recognized as an effective permeation enhancer in transdermal drug delivery^75,76^ and its interactions with the stratum corneum have been extensively investigated.^77−79^ However, our findings confirmed its specific influence when incorporated into the release medium used for IVPT studies. While the skin integrity test affirmed media suitability, the rise in TEWL suggests that ethanol in the release medium may compromise the skin’s integrity, accelerating LOR permeation following its release from MAPs, possibly due to lipid structure disruption.^79^ In addition to the impairment of skin integrity, a higher drug solubility in the medium can create a steeper concentration gradient, leading to more rapid diffusion through the skin and ultimately resulting in a higher drug amount quantified in the release medium. While ethanol markedly enhances drug solubility, this effect may not faithfully reflect physiological conditions. Using a medium that closely resembles body fluids might be preferable over one solely providing sink conditions. Yet, this approach poses a practical challenge in accurately measuring drug quantities in the medium. Furthermore, depending on the route of drug administration, the human body may not always act as a sink for drugs. Hence, finding the right balance between the drug solubility and physiological conditions expected *in vivo* is crucial for an accurate prediction of drug behavior. Although the application of the AIF in IVPT offers a closer approximation to *in vivo* conditions, establishing sink conditions for poorly soluble drugs proves to be challenging without the inclusion of a surfactant. It is well-known that ionic surfactants like sodium lauryl sulfate (SLS) or cetyltrimethylammonium bromide have a greater potential to damage the skin, while nonionic surfactants like PEG-400 and polysorbate-80 exert a milder effect,^57^ which aligns with the findings of our study.

Given that PBS is frequently employed for IVPT studies involving hydrophilic drugs, we investigated CPM permeation behavior when using both PBS and AIF. While an evident similarity in the trend of drug permeation was observed, it is noteworthy that PBS exhibited a consistently higher cumulative amount permeated at each time point than AIF (Figure 8a). This disparity cannot be attributed to the solubility differences, as both the drug solubility and release profile in PBS and AIF remain comparable. Since the composition of PBS and AIF is different (Table S1, Supporting Information), the slower permeation observed in AIF suggests the possibility of interactions between the skin and AIF, potentially influencing permeation kinetics. Considering that AIF has not been previously explored in the context of topical and transdermal *in vitro* studies, there is a clear need for further research to investigate the interactions between AIF and the skin. Given the prevalent use of PBS in *in vitro* studies, it becomes imperative to acknowledge the divergence between this commonly used medium and the biorelevant *in vivo* environment resembling AIF. Consequently, the potential overprediction of outcomes derived from *in vitro* studies extrapolating to *in vivo* conditions must be considered for both lipophilic and hydrophilic drug molecules. Although this study specifically focused on the influence of the *in vitro* release media composition, further investigation into other experimental variables (e.g., skin preparation methods, skin models, and skin metabolism) is necessary to enhance IVPT for MAPs.

## 5 Conclusions

As shown in this study, IVRT combined with IVPT represents a multifaceted approach for the evaluation of drug-loaded MAP performance. We have demonstrated the significant impact of *in vitro* release media composition on drug release from MAPs and its subsequent permeation into the release medium. Study findings highlight release medium choice as a key consideration in the design of IVRT and IVPT methodologies to ensure accurate representation of the complex interplay between the drug, the delivery system, and the skin. Additionally, the findings emphasize the need for caution when incorporating specific excipients to enhance the solubility of poorly soluble compounds. This becomes particularly important considering the potential interactions these excipients may have with the skin. The multifaceted IVRT and IVPT approach has the potential to move beyond merely being quality assessments for MAP drug delivery systems to offer a more accurate reflection of *in vivo* performance. However, further research is required to establish correlations with biorelevant release media and *in vivo* conditions as well as to tailor the approach for drugs with diverse physicochemical properties.

## Supporting Information Available

Table S1: Composition of artificial interstitial fluid; Table S2: DoE experimental runs – full factorial design for loratadine skin extraction; Table S3: DoE experimental runs – full factorial design for chlorpheniramine maleate skin extraction; Figure S1: Pareto chart illustrating main effects of factors on the percentage of loratadine extraction from the skin using full factorial design; Figure S2: Pareto chart illustrating main effects of factors on the percentage of chlorpheniramine maleate extraction from the skin using full factorial design; Figure S3: Average amount of loratadine in the skin and on MAP after 24 h; Figure S4: Average amount of chlorpheniramine maleate in the skin after 24 h (PDF)
