# Supplementary material for: Unravelling Microarray Patch Performance: The Role of In Vitro Release Medium and Biorelevant Testing
Source: Mol Pharm. 2024 Aug 28;21(10):5028–40. doi: 10.1021/acs.molpharmaceut.4c00459 (PMC11462508; doi:10.1021/acs.molpharmaceut.4c00459)
Supplement: Supplementary file 1 — mp4c00459_si_001.pdf [file mp4c00459_si_001.pdf]

Supporting Information: Additional experimental details and results including media composition, DoE runs and drugs' amount in the skin

Unravelling Microarray Patch Performance: The Role of *In Vitro* Release Medium and Biorelevant Testing

Maja Railic, Abina M. Crean, Sonja Vucen*

SSPC, the SFI Research Centre for Pharmaceuticals, School of Pharmacy, University College Cork, College Road, Cork, T12 K8AF, Ireland

*Email: svucen@ucc.ie

Table S1: Composition of Artificial interstitial fluid (AIF)

Component	Concentration (mmol/L)
NaCl	107.7
KCl	3.5
CaCl2	1.53
MgSO4	0.69
NaHSO4	26.2
NaH2PO4	1.67
NaC6H11O7	9.64
Glucose	5.55
Sucrose	7.6

Table S2: DoE experimental runs – full factorial design for loratadine skin extraction. ACN- Acetonitrile; URT- Uncontrolled room temperature

Run number	Solvent	Temperature	Number of homogenization cycles
1	Methanol	URT	1
2	Methanol:ACN (70:30)	URT	1
3	Methanol	55°C	1
4	Methanol:ACN (70:30)	55°C	1
5	Methanol	URT	2
6	Methanol:ACN (70:30)	URT	2
7	Methanol	55°C	2
8	Methanol:ACN (70:30)	55°C	2
9	Methanol	URT	1
10	Methanol:ACN (70:30)	URT	1
11	Methanol	55°C	1
12	Methanol:ACN (70:30)	55°C	1
13	Methanol	URT	2
14	Methanol:ACN (70:30)	URT	2
15	Methanol	55°C	2
16	Methanol:ACN (70:30)	55°C	2

Table S3: DoE experimental runs – full factorial design for chlorpheniramine maleate skin extraction.

Run number	Solvent	Additional grinding step
1	Methanol:Water (80:20)	No
2	Propanol:Water (70:30)	No
3	Methanol:Water (80:20)	No
4	Methanol:Water (80:20)	Yes

5	Ethanol:Water (70:30)	No
6	Methanol:Water (70:30)	Yes
7	Propanol:Water (70:30)	Yes
8	Propanol:Water (70:30)	No
9	Propanol:Water (70:30)	Yes
10	Ethanol:Water (70:30)	Yes
11	Ethanol:Water (70:30)	Yes
12	Ethanol:Water (70:30)	No

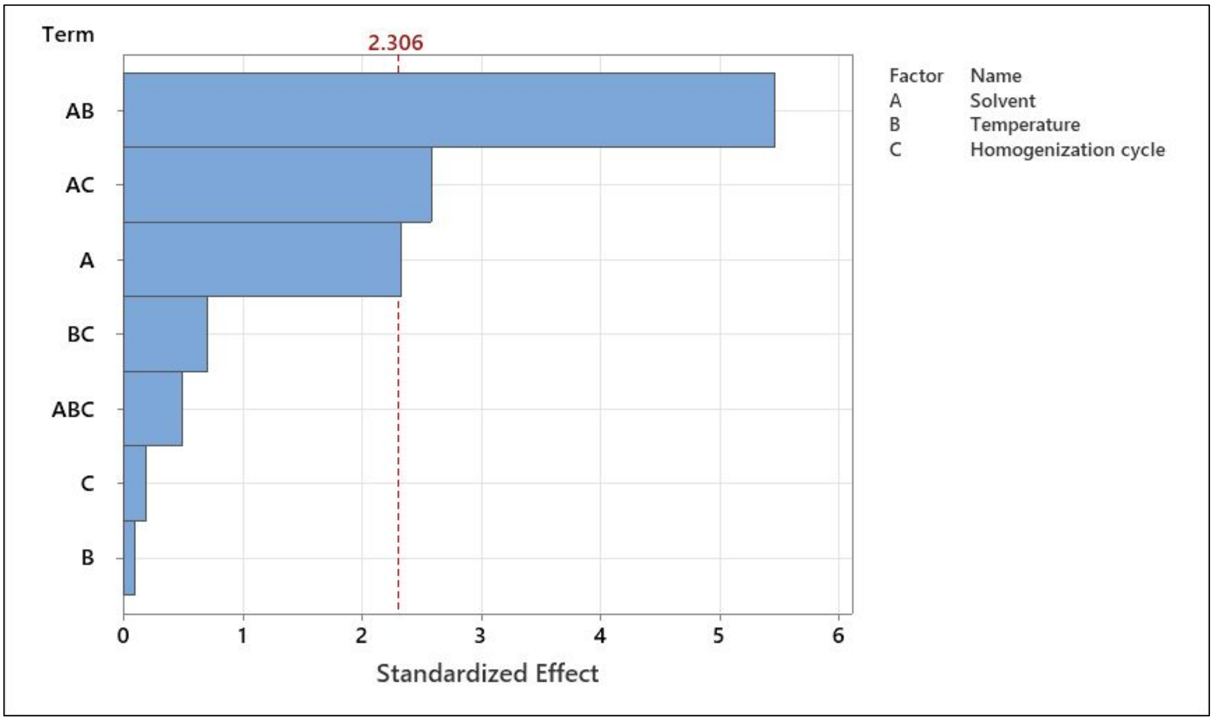

Figure S1: Pareto chart illustrating main effects of factors on the percentage of loratadine extraction from the skin using full factorial design (a=0.05).

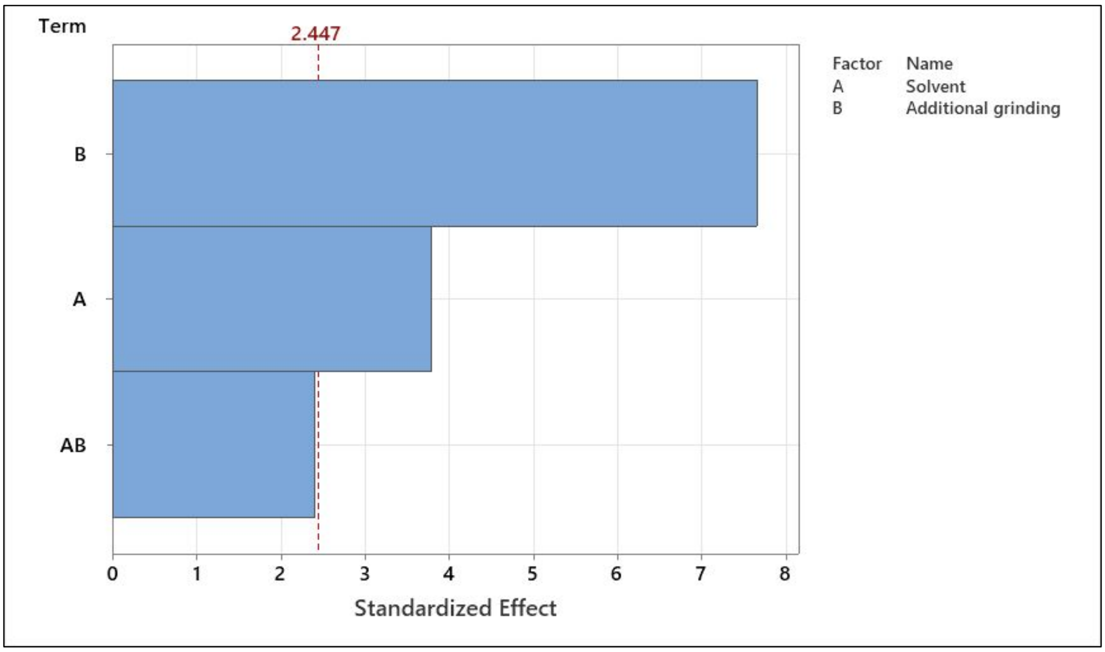

Figure S2: Pareto chart illustrating main effects of factors on the percentage of chlorpheniramine maleate extraction from the skin using full factorial design (a=0.05)

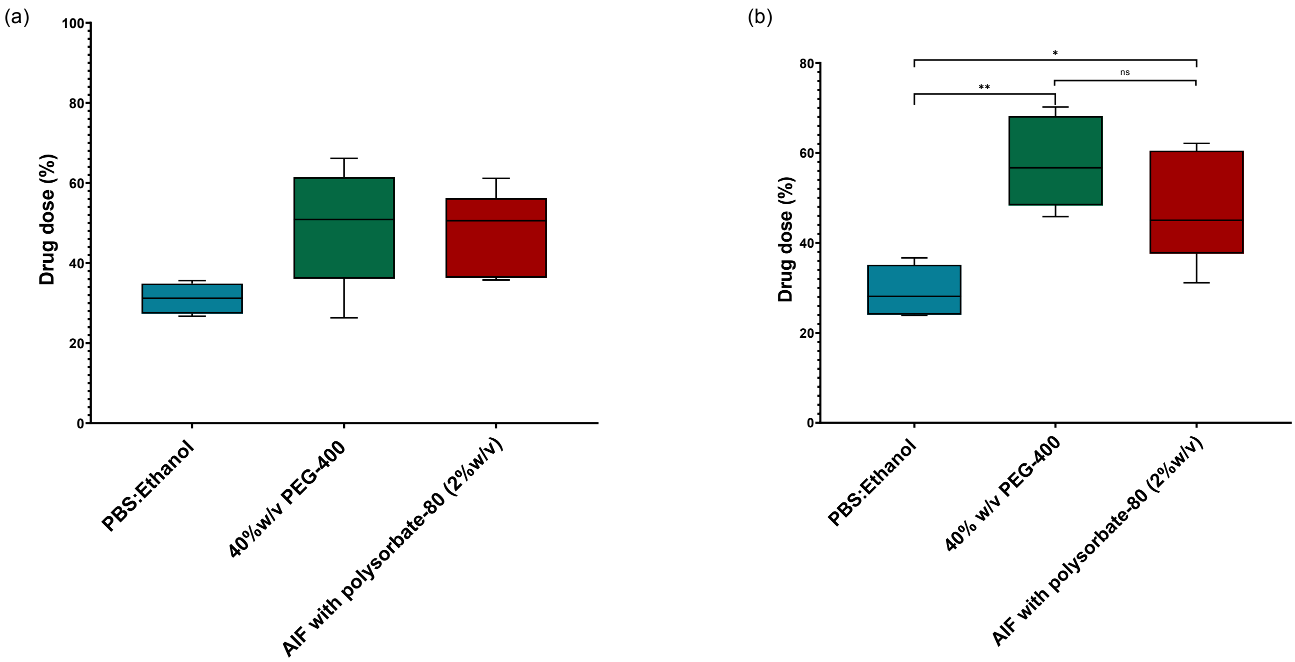

Figure S3: Average amount of loratadine in the skin (a) and on MAP (b) after 24-hour IVPT with different release media (n=5) (one-way ANOVA). No significant differences in drug content within the skin were observed among the three release media conditions (p=0.0553, two-way ANOVA). Asterisks denote statistical

significance on the tape: * p ≤ 0.05, ** p ≤ 0.01. "ns" denotes no significant difference. Y error bars indicate standard deviation.

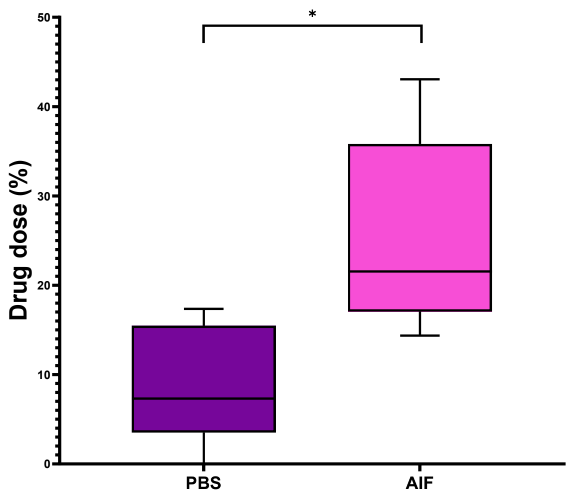

Figure S4: Average amount of chlorpheniramine maleate in the skin after 24-hour IVPT with different release media (n=5) (unpaired t-test). The * denotes statistical significance ($p \leq 0.05$). Y error bars indicate standard deviation.
